# Antioxidant enzymes activities in obese Tunisian children

**DOI:** 10.1186/1475-2891-12-18

**Published:** 2013-01-29

**Authors:** Sonia Sfar, Raoudha Boussoffara, Mohamed Tahar Sfar, Abdelhamid Kerkeni

**Affiliations:** 1 Unité de recherche «Eléments trace, radicaux libres, systèmes antioxydants et pathologies humaines», Faculté de médecine de Monastir, Université de Monastir, rue Avicenne, 5019, Monastir, Tunisia; 2 Service de Pédiatrie, Hôpital universitaire de Mahdia, 5111, Mahdia, Tunisia

**Keywords:** Catalase, Glutathione peroxidase, Superoxide dismutase, Obesity, Oxidant stress, Children

## Abstract

**Background:**

The oxidant stress, expected to increase in obese adults, has an important role in the pathogenesis of many diseases. It results when free radical formation is greatly increased or protective antioxidant mechanisms are compromised. The main objective of this study is to evaluate the antioxidant response to obesity-related stress in healthy children.

**Methods:**

A hundred and six healthy children (54 obese and 52 controls), aged 6–12 years old, participated in this study. The collected data included anthropometric measures, blood pressure, fasting glucose, total cholesterol, triglycerides and enzymatic antioxidants (Superoxide dismutase: SOD, Catalase: CAT and Glutathione peroxidase: GPx).

**Results:**

The first step antioxidant response, estimated by the SOD activity, was significantly higher in obese children compared with normal-weight controls (p < 0.05). Mean activities of anti-radical GPx and CAT enzymes were not affected by the BMI increase. Although, total cholesterol levels were statistically higher in the obese group, there was no significant association with the SOD activity.

**Conclusions:**

The obesity-related increase of the oxidant stress can be observed even in the childhood period. In addition to the complications of an increased BMI, obesity itself can be considered as an independent risk factor of free radical production resulting in an increased antioxidant response.

## Background

Obesity has become a major health problem in both developed and under-developed countries. It is a complex disease that involves the interactions between environmental [1] and genetic factors [2]. The prevalence of obesity has increased at an alarming rate during the last three decades, and has been particularly problematic in children [3]. The main direct adverse effects of childhood obesity include orthopedic complications, sleep apnea, and psychological disorders [4]. Approximately 70% of obese adolescents grow up to become obese adults [5].

Defining the basic physiopathology of childhood obesity should be the first step in preventing the associated consequences that may develop lifelong. In particular, the cellular defense mechanisms and their influence against many diseases were thought to be related to obesity and its complications as seen in adults [6]. Cells have developed an enzymatic antioxidant pathway against reactive oxygen species (ROS) that are generated during oxidative metabolism: first, the dismutation of superoxide anion (O_2_^-^) to hydrogen peroxide (H_2_O_2_) catalyzed by superoxide dismutase (SOD); and then, the conversion of H_2_O_2_ to H_2_O by glutathione peroxidase (GPx) or catalase (CAT). The activity of first- and second-step antioxidant enzymes must be balanced to prevent oxidative damage in cells, which may contribute to various pathological processes [7]. Compared to adult studies, the knowledge about the response to oxidant stress damage caused by obesity during childhood is limited.

The present work aimed at investigating the effect of the childhood obesity on the antioxidant enzyme activities (SOD, GPx and CAT).

## Methods

### Subjects

The study population consisted of 106 healthy children, aged between 6 and 12 years old: 54 exogenic obese children (31 girls and 23 boys) and 52 controls (25 girls and 27 boys). The recruitment was performed between January 2012 and March 2012, from 25 elementary schools in the district of Mahdia (eastern central coast of Tunisia), which included a mixture of rural and urban environments. Obesity diagnosis was obtained according to the body mass index scale, and a percentile more than 95% was considered [8,9]. Endogenic obesity (hypothyroidism, Cushing’s syndrome) and genetic syndromes (Alström, Bardet-Biedl) were ruled out by physical examination and laboratory tests. Chronic diseases like diabetes, hepatitis, epilepsy, and renal diseases, were included in the exclusion criteria. The control group was selected from healthy children without any complaint and normal physical examination with normal laboratory findings. Taking into account gender- and age-related variations of children growth rate (in weight, height and body mass index) [9], both gender populations were divided into two groups: 6–8.5 and 9–12 years old.

Age, sex, weight including light clothing (±0.1 kg), height without shoes (±0.1 cm) and body mass index (weight/height^2^) of all children were recorded. Waist and hip circumferences were measured using a tape measure to the nearest 1 mm. Waist circumference was measured midway between the lowest rib and the superior border of iliac crest at the end of normal expiration. Hip circumference was measured at the level of widest portion of buttocks (trochanters). Blood pressure was measured using a mercury sphygmomanometer. The measurements were done after 30 min of resting, on both arms. The highest value of pressure was considered for every child. The children were carefully instructed to fast for a period of at least 12 h. A parental consent was obtained from every child included in the study population. The local ethical committee of the hospital of Mahdia approved the study protocols.

### Blood samples collection

A venous blood sample was obtained from every fasting volunteer into heparinized tubes (BD Vacutainer System). All the blood samples were immediately carried to the laboratory of the academic hospital of Mahdia in a crushed ice block. Erythrocytes and plasma were separated by centrifugation at 3600 g for 10 min, aliquoted, coded, frozen and stored at −20°C until analysis. In addition to the assays described below, other biochemical measurements including fasting plasma glucose, plasma total cholesterol and plasma triglycerides were performed.

### Antioxidant enzymes activity

The activity of anti-radical enzymes, namely the superoxide dismutase (SOD), the catalase (CAT), and the glutathione peroxidase (GPx) was determined by spectrophotometry (spectrophotometer Shimadzu 160 A). Hemoglobin concentration was estimated in every blood sample from the absorbance measurement at 546 nm (The colorimetric method). Set solutions were prepared using 20 μL from 1/10 diluted erythrocytes and 1 mL from diluted Drabkin solution (0.77 mM KCN, 0.6 mM K_3_(Fe(CN)_6_) and 0.01 M K(H_2_PO_4_)).

The SOD activity was determined in erythrocytes using pyrogallol as a substrate by the method of Marklund and Marklund [10]. This method is based on pyrogallol oxidation by the superoxide anion (O_2_^-^) and its dismutation by SOD enzyme. Red blood cells were first hemolysed with cold distilled water. After extraction with ethanol/chloroform mixture (1:1) followed by centrifugation at 3600 rpm at 4°C during 25 min, the organic phase was collected. A mixture (935 μL of Tris tampon and 40 μL of pyragollol) was then added to 25 μL of the (1/2) diluted organic phase solution. The enzymatic activity, in units per milligrams of hemoglobin (U/mg Hb), was measured in the supernatant at 420 nm according to Winterbourn et al. [11]. A unit of SOD activity corresponds to the least enzyme quantity able to inhibit the auto-oxidation reaction of the added pyragollol at 50%. Results were expressed as U(SOD)/mg Hb.

CAT activity (U/g Hb) was estimated in erythrocytes according to the method of Beers and Sizer [12] using hydrogen peroxide as a substrate. The method is based on the decomposition of hydrogen peroxide which is indicated by the decrease in absorbance at 240 nm [13]. To obtain the assay solution, 2.9 mL of phosphate buffer solution were therefore added to 100 μL of hemolyzed red cells. The absorbance of the obtained mixture was then measured during 1 min. Consumption of 3.45 μmol hydrogen peroxide is characterized by (0.45–0.4) absorbance decrease.

The activity of GPx (U/g Hb) was assayed in erythrocytes by the method of Günzler et al. using t-butyl hydroperoxide (tBOOH) as a substrate [14]. After dilution, 25 μL of hemolyzed red cells were added to 900 μL of buffer Tris, 20 μL of glutathione (GSH), 20 μL of glutathion reductase (GSH-Rase) and 20 μL of NADPH2. Twenty microliters of tBOOH were then added and finally, decrease of NADPH2 optical density was followed up at 340 nm.

Coefficients of variation (CV) were 2.9%, 3.5% and 4.9% for SOD, CAT and GPx activities. An in-house pool of human erythrocytes was used as interval quality control.

### Statistical analysis

The statistical analysis was performed using the SPSS package (12.0 for Windows). The results are presented as means ± SD. As recommended in [15], the blood pressure z-scores were calculated in reference to the height. Differences in mean values were evaluated using the one-way analysis of variance (ANOVA). Each variable was examined for normal distribution using the Shapiro-Wilk test. The Pearson’s model was used to test associations between the considered variables. Statistical significance was considered at p < 0.05.

## Results

Mean variations of anthropometrical data are presented in Table 1. For both gender, the study population has been separated in two groups according to age interval: 6–8.5 and 9–12 years old. The weight, the height and then the BMI are expected to show important age-related variations during childhood and adolescence [9]. Note that the subject group size (n) is nearly the same according to gender and age intervals: The boys/girls ratio is 50:56. The 6–8.5 years and 9–12 years obese/controls ratios are respectively 11:12 and 12:15 (for boys), 13:11 and 18:14 (for girls). For all the children groups, there is no significant difference in mean age between obese and normal-weight volunteers (Table 1). No variation in mean height is noteworthy, except for girls aged 9–12 years old (151 ± 0.1 cm and 141.5 ± 0.09 cm for obese and normal-weight girls respectively, p < 0.01). However, mean BMI is shown to be considerably higher in obese children than in controls for all age groups. Significant differences are also obtained in weight mean value for all subject groups. In addition, obese children have higher waist and hip circumferences than normal-weight subjects (p < 0.05). Differences in diastolic and systolic blood pressure according to BMI classification are not significant. Similarly, systolic and diastolic z-scores referred to the height show no significant variation between obese and normal-weight children. Plasma total cholesterol is shown to significantly increase in obese children (p < 0.01 and p < 0.001 respectively for 6–8.5 and 9–12 years age intervals). Other fasting biochemical levels including plasma glucose and triglycerides do not show any significant difference between obese and normal-weight children (Table 1). The same remark is available for age- and gender- related variations. For all subgroups, the obtained mean data show a good concordance with the reference values according to the European recommendation guidelines. This confirms the normal health status of the recruited children.

**Table 1:** General characteristics of the study population (mean ± SD)

	**Boys (n=50)**				**Girls (n=56)**			
	**Age: 6–8.5 years**		**Age: 9–12 years**		**Age: 6–8.5 years**		**Age: 9–12 years**	
	**Obese children (n=11)**	**Controls (n=12)**	**Obese children (n=12)**	**Controls (n=15)**	**Obese children (n=13)**	**Controls (n=11)**	**Obese children (n=18)**	**Controls (n=14)**
Age (years)	7.38 ± 0.86	7.39 ± 0.85	11.36 ± 0.67	11.28 ± 1.32	7.55 ± 0.92	7.44 ± 0.90	11.06 ± 1.24	11.29 ± 0.48
Height (cm)	131.0 ± 0.1	128.6 ± 0.1	151.6 ± 0.06	146.1 ± 0.12	129.0 ± 0.06	127.0 ± 0.07	151.0 ± 0.1 ^a^	141.5 ± 0.09
Weight (kg)	37.43 ± 6.37 ^b^	25.86 ± 4.02	63.90 ± 5.61 ^a^	36.07 ± 8.38	37.83 ± 7.22 ^a^	26.67 ± 3.56	62.47 ± 12.72 ^b^	33.33 ± 6.10
BMI (kg/m^2^)	22.28 ± 2.46 ^b^	15.41 ± 0.98	28.57 ± 4.28 ^b^	17.03 ± 1.87	23.24 ± 2.45 ^b^	15.81 ± 1.37	27.44 ± 2.84 ^b^	16.14 ± 2.10
Waist circumference (cm)	68.33 ± 4.23 ^a^	54.5 ± 2.12	99.0 ± 4.14 ^b^	61.83 ± 5.74	77.2 ± 3.83 ^b^	53.67 ± 2.31	87.67 ± 8.45 ^a^	65.0 ± 8.18
Hip circumference (cm)	76.5 ± 7.31 ^a^	63.0 ± 1.41	98.5 ± 6.26 ^a^	69.5 ± 5.28	84.25 ± 6.85 ^a^	60.33 ± 4.2	99.67 ± 9.46 ^a^	75.33 ± 5.77
Systolic BP (mm Hg)	125.0 ± 5.9	102.5 ± 3.5	124 ± 5.7	104.5 ± 5.8	127.5 ± 6.5	106.7 ± 5.8	129.8 ± 8.0	118.3 ± 7.6
Systolic BP z-score	1.28 ± 0.68	1.11 ± 0.88	2.88 ± 1.1	2.81 ± 1.2	1.85 ± 0.82	1.38 ± 0.53	2.83 ± 0.61	2.27 ± 0.47
Diastolic BP (mm Hg)	66.7 ± 5.2	65.7 ± 7.5	72.5 ± 5.6	67.5 ± 2.7	68.8 ± 7.5	63.3 ± 5.8	72.5 ± 5.4	70.0 ± 6.7
Diastolic BP z-score	0.39 ± 0.19	0.29 ± 0.16	0.88 ± 0.18	0.82 ± 0.21	0.54 ± 0.18	0.46 ± 0.1	1.09 ± 0.46	0.78 ± 0.37
Glucose* (mmol/L)	3.86 ± 0.56	3.97 ± 0.51	4.24 ± 0.92	4.42 ± 0.71	3.70 ± 0.50	4.04 ± 0.45	4.57 ± 0.55	4.45 ± 0.73
Total cholesterol** (mmol/L)	4.22 ± 1.21 ^a^	3.13 ± 0.97	4.39 ± 0.79 ^b^	3.05 ± 0.41	4.29 ± 1.26 ^a^	3.18 ± 1.17	4.40 ± 0.31 ^b^	3.10 ± 0.21
Triglycerides*** (mmol/L)	0.95 ± 0.15	0.85 ± 0.14	0.98 ± 0.12	0.82 ± 0.18	0.92 ± 0.15	0.79 ± 0.17	0.94 ± 0.18	0.82 ± 0.17

Normal fasting ranges (according to the European guidelines): * 3.3 − 6 mmol/L; ** 4–6 mmol/L; *** 0.6 − 1.7 mmol/L.

*BP* Blood pressure.

^a^ (p < 0.01); ^b^ (p < 0.001).

Mean anti-radical enzyme activities according to gender and age interval are summarized in Table 2. The activity of the first-step antioxidant enzyme (SOD) is shown to increase in association with pediatric obesity. For both gender, the SOD activity is significantly higher in obese children than in normal-weight subjects for every age interval. Girls show relatively higher antioxidant SOD activity in comparison with boys (p < 0.01). The mean activities of the second-step anti-radical enzymes (GPx and CAT) do not show any significant difference between obese and normal-weight children in all gender and age-related groups. No significant gender-associated difference is obtained in GPx and CAT activities.

**Table 2:** The mean anti-radical enzyme activities (GPx, SOD and CAT) in obese and normal -weight children (mean ± SD)

	**Boys**				**Girls**			
	**Age: 6–8.5 years**		**Age: 9–12 years**		**Age: 6–8.5 years**		**Age: 9–12 years**	
	**Obese children (n=11)**	**Controls (n=12)**	**Obese children (n=12)**	**Controls (n=15)**	**Obese children (n=13)**	**Controls (n=11)**	**Obese children (n=18)**	**Controls (n=14)**
SOD (U/mg Hb)	3.37 ± 0.96 ^*^	2.59 ± 0.91	3.41 ± 1.05 ^**^	2.75 ± 1.03	3.92 ± 0.79 ^*^	3.08 ± 0.92	4.31 ± 0.91 ^**^	3.35 ± 0.78
GPx (U/g Hb)	39.77 ± 6.98	32.48 ± 7.29	34.38 ± 8.52	33.83 ± 8.9	38.83 ± 7.26	34.41 ± 6.22	36.55 ± 6.48	35.64 ± 8.31
CAT (U/g Hb)	32.48 ± 5.95	30.83 ± 8.63	32.15 ± 7.27	29.22 ± 7.39	32.69 ± 8.44	31.65 ± 6.54	39.77 ± 12.25	38.27 ± 11.84

*: p < 0.01; **: p < 0.05.

Table 3 shows the correlation coefficients among GPx, SOD and CAT activities, and various parameters (age, BMI, waist and hip circumferences, glucose, total cholesterol and triglycerides). SOD is found to be significantly and positively associated with BMI using the Pearson rank correlation: r = 0.3 (p = 0.04) and r = 0.15 (p = 0.036) respectively for boys and girls. Associations between the activity of SOD and the remaining parameters are not significant. Variations of GPx and CAT activities according to BMI are not relevant. No significant association is obtained between GPx, SOD or CAT activity, with age, waist and hip circumferences, glucose, total cholesterol and triglycerides serum fasting levels. Partial correlation test adjusted by age has been also performed. The obtained results show that only the SOD activity is significantly correlated with the BMI, for girls (r = 0.19, p < 0.01) and for boys (r = 0.32, p = 0.05).

**Table 3:** Correlation coefficients among age, BMI, biochemical levels, and antioxidant enzyme activities

		**Boys (n=50)**			**Girls (n=56)**		
		**GPx**	**SOD**	**CAT**	**GPx**	**SOD**	**CAT**
Age	Correlation coefficient	−0.04	−0.12	−0.22	−0.12	0.13	−0.04
	Significance	0.8	0.52	0.21	0.43	0.45	0.79
BMI	Correlation coefficient	0.17	0.3*	−0.08	−0.03	0.15*	−0.08
	Significance	0.28	0.04	0.65	0.87	0.036	0.57
Waist circumference	Correlation coefficient	−0.05	0.18	−0.11	−0.23	0.03	0.11
	Significance	0.86	0.58	0.69	0.39	0.91	0.71
Hip circumference	Correlation coefficient	0.07	0.33	−0.11	−0.29	0.14	0.19
	Significance	0.81	0.28	0.69	0.28	0.62	0.51
Glucose	Correlation coefficient	0.19	0.06	0.18	−0.23	−0.27	−0.18
	Significance	0.41	0.83	0.48	0.26	0.16	0.43
Total cholesterol	Correlation coefficient	0.32	0.14	0.15	0.36	0.23	0.12
	Significance	0.28	0.35	0.19	0.16	0.52	0.26
Triglycerides	Correlation coefficient	−0.03	0.18	−0.05	0.07	0.19	−0.42
	Significance	0.69	0.19	0.56	0.37	0.29	0.13

* Significant correlation (p < 0.05).

## Discussion

The relationship between obesity and oxidant stress parameters has been extensively studied in healthy adults. However, it is unknown whether this relationship exists from early age and if it has the same trends during childhood. The response to oxidant damage caused by ROS production differs from age to age: The maturation of antioxidant enzymes is, in fact, related to ageing. In this study, we examined the variations of antioxidant enzyme activities – considered biomarkers of oxidant stress – in a population of healthy obese children. Cases of endogenic obesity were not considered to exclude other factors influencing the antioxidant enzyme activity [16]. A control group composed of healthy normal-weight children was established with comparable age and gender-related distributions. Obesity indicators, such as BMI, waist and hip circumferences, were shown to significantly increase in diagnosed obese children. There was also no gender disparity in BMI, waist or hip circumference, in line with previous studies [16-18].

Obesity can be defined as an excess of body fat. There are several methods of measuring the percentage of body fat. In the clinical environment, techniques based on BMI, waist or hip circumference, and skin fold thickness have been extensively used and suggested to be satisfactory in identifying risk [19]. Some authors [20] have suggested using waist circumference since it seems to be more accurate for children: it targets central obesity, which is a risk factor for type II diabetes and coronary heart disease.

This study showed that the antioxidant enzyme activity of SOD was markedly higher in the obese children compared to the normal-weight ones, taking into account the gender and the age-related variations. No significant difference was obtained in the second step enzymes (GPx and CAT) activities. These results confirm the fact that obesity is associated with the oxidant stress increase, even in early age. Possible mechanisms contributing to the obesity-associated oxidant stress include increased oxygen consumption and subsequent radical production via mitochondrial respiration, increased fat deposition and cell injury causing increased rates of radical formation [21]. The cell adaptation to the increase of radical production, as a consequence of obesity, consists then in the increase of SOD activity. In pediatric obesity, only the first-step enzyme (SOD) activity seems to be affected by the increase of body fat. A previous investigation performed on healthy children had also shown that the SOD activity was enhanced in association to obesity [16]. Other studies on animal and human adults showed controversial results. Koboyasi et al. [22] demonstrated that the production of superoxide anion and the Cu/ZnSOD activity increased in obese mice. Nakao et al. [23] also reported significant increased SOD levels in the liver, kidney, muscles, plasma, and white and brown adipose tissues of obese mice. Vincent et al. [21] found that the Cu/ZnSOD activity in the left ventricles of rats was greater in the obese animals compared to lean controls. Other studies have identified no significant or opposite difference in individual antioxidant enzyme concentrations in obesity. Brown et al. [6] found no significant difference in total antioxidant status, SOD and reduced glutathione among normal-weight, overweight, and obese adults. On the contrary, Olusi [24] found that erythrocyte Cu/ZnSOD activity was significantly lower in obese subjects compared to results from control subjects. Similarly, Ozata et al. [25] reported 42% lower SOD activity in obese vs. non-obese men. These discrepancies could be linked to the duration of obesity. For example, in the development stages of obesity, antioxidant enzymes may be stimulated whereas in chronic and long-term obesity, the sources of antioxidant enzymes become depleted, leading to a low level activity [6]. An example of antioxidant stimulation during the development stages of obesity has been shown in the study of Dobrian et al. [26] who reported increases in the activity of SOD after 10 weeks of diet-induced obesity in rats. The effects of chronic obesity has not been scientifically studied but could be achieved by studying obese individuals of similar weight and body composition and varying degrees of length of obesity time.

The anti-oxidant enzyme activity is subject to various stress-related factors, including physical activity and hypercholesterolemia. Woo et al. [27] investigated the effects of detraining on the antioxidant enzyme in Korean overweight children. They showed, in particular, that the protein and expression of GPx was increased following a 12-week as compared with prior to training. The protein and expression of SOD showed no significant changes. On the other hand, it had been shown that hypercholesterolaemic non-obese children had lower red cell antioxidant enzymes [28]. As a consequence, they could be more susceptible to the adverse effects of reactive oxygen species. In the present study, these factors have not been considered to investigate separately the effect of obesity on the markers of the antioxidant enzyme activity.

In addition, hyperglycemia, hypertension, and hypercholesterolemia are also possible sources of increased oxidant stress in the obese state [29]. The present study showed that the antioxidant enzyme defense was enhanced in obese children, which had normal health parameters including blood pressure, glucose, and triglycerides concentrations. Moreover, the associations between the antioxidant enzyme activities and these biochemical data were not significant. This allows rejecting the hypothesis saying that the obesity-associated oxidant stress is only a consequence of obesity-related diseases [30,31]. Instead, it can be related to the excess in the adipose tissue accumulation. These findings are also confirmed by Keaney et al. [32] who found in a community-based cohort, a strong association between markers of oxidant stress and BMI, implicating adiposity as the main factor for increased oxidant stress.

When the plasma levels of total cholesterol of obese and normal-weight children were compared, there was a significant increase in the obese group. Although, both total cholesterol and SOD levels were significantly higher in obese children, no correlation was observed between the two parameters. Free radicals are very potent oxidants and react with lipids to result in lipid peroxidation [33].

Although, the general consensus is that obesity (and increased BMI) increases oxidant stress even during childhood, it is difficult to identify a typical-related oxidant stress response because the majority of studies have incorporated different methodology designs including different oxidant stress markers.

## Conclusions

Obesity is still a major health problem in most countries, particularly in children. Then, defining the basic physiopathology of childhood obesity should be the first step in preventing the associated consequences that may develop lifelong. In particular, it is still unknown whether the cellular defense mechanisms and their influence against many diseases are widely related to obesity and its complications. This study investigated the effect of obesity on the antioxidant body response in healthy children. In support of previous research findings, the oxidant stress resulting from obesity appeared at an early age. In fact, the activity of the first-step enzyme SOD was shown to increase in association with the increased BMI. In addition to obesity-related complications such as hyperlipidemia and diabetes mellitus, obesity itself is an independent risk factor in the matter of free radicals which are known to be responsible for organ damage in many diseases. It would be interesting to identify and test dietary components that can catch or prevent the excess in the production of the reactive oxygen radicals using both clinical and experimental investigations. Other potential research studies may identify suitable strategies to reverse the obesity-associated oxidant stress (weight loss, calorie restriction, exercise training, etc.).

## Abbreviations

SOD: Superoxide dismutase; GPx: Glutathione peroxidase; CAT: Catalase; BMI: Body mass index; ROS: Reactive oxygen species.

## Competing interests

The authors declare that they have no competing interests.

## Authors’ contributions

SS Conceived the study, and participated in its design and coordination, performed the statistical analysis, drafted the original manuscript and edited the final paper. RB participated in the design and coordination of the study. MTS provided key insight into the conception and the coordination of the study, and contributed to the final review of the manuscript. AK participated in the coordination of the experimental part of the study. All authors read and approved the final manuscript.
